# Karyotype Analysis of Diploid and Spontaneously Occurring Tetraploid Blood Orange [*Citrus sinensis* (L.) Osbeck] Using Multicolor FISH With Repetitive DNA Sequences as Probes

**DOI:** 10.3389/fpls.2019.00331

**Published:** 2019-03-22

**Authors:** Honghong Deng, Zexi Cai, Suqiong Xiang, Qigao Guo, Wei Huang, Guolu Liang

**Affiliations:** ^1^College of Horticulture and Landscape Architecture, Southwest University, Chongqing, China; ^2^College of Agronomy and Biotechnology, National Maize Improvement Center, China Agricultural University, Beijing, China

**Keywords:** multicolor fluorescence *in situ* hybridization, satellite DNA, rDNA, chromosome identification, karyotype, tetraploidization

## Abstract

Blood orange [*Citrus sinensis* (L.) Osbeck] has been increasingly appreciated by consumers worldwide owing to its brilliant red color, abundant anthocyanin and other health-promoting compounds. However, there is still relatively little known about its cytogenetic characteristics, probably because of the small size and similar morphology of metaphase chromosomes and the paucity of chromosomal landmarks. In our previous study, a naturally occurring tetraploid blood orange plant was obtained via seedling screening. Before this tetraploid germplasm can be manipulated into a citrus triploid seedless breeding program, it is of great importance to determine its chromosome characterization and composition. In the present study, an integrated karyotype of blood orange was constructed using sequential multicolor fluorescence *in situ* hybridization (FISH) with four satellite repeats, two ribosomal DNAs (rDNAs), a centromere-like repeat and an oligonucleotide of telomere repeat (TTTAGGG)_3_ as probes. Satellite repeats were preferentially located at the terminal regions of the chromosomes of blood orange. Individual somatic chromosome pairs of blood orange were unambiguously identified by repetitive DNA-based multicolor FISH. These probes proved to be effective chromosomal landmarks. The karyotype was formulated as 2*n* = 2*x* = 18 = 16m+2sm (1sat) with the karyotype asymmetry degree belonging to 2B. The chromosomal distribution pattern of these repetitive DNAs in this spontaneously occurring tetraploid was identical to that of the diploid, but the tetraploid carried twice the number of hybridization sites as the diploid, indicating a possible pathway involving the spontaneous duplication of chromosome sets in nucellar cells. Our work may facilitate the molecular cytogenetic study of blood orange and provide chromosomal characterization for the future utilization of this tetraploid germplasm in the service of seedless breeding programs.

## Introduction

As one of the most important citrus crops, blood (or red) orange [*Citrus sinensis* (L.) Osbeck, 2*n* = 2*x* = 18] (Krug, 1943), is extensively cultivated in some Mediterranean basin (Italy and Spain), the United States (CA), Australia, and China, and it is consumed worldwide as fresh fruit and a processed juice product (Caruso et al., 2016). In particular, blood orange has become a mainstay and hallmark of the citrus industry in Sicily (southern Italy). Taxonomically, the blood orange is a spontaneous bud mutation of a sweet orange variety with crimson flesh (Butelli et al., 2012). This pigmented variety has been increasingly appreciated by consumers worldwide, owning to its pleasant taste, brilliant red flesh color and abundant health-promoting compounds (Butelli et al., 2012; Grosso et al., 2013; Caruso et al., 2016). Blood orange fruits are rich in antioxidants, especially naturally occurring phenolic compounds, including anthocyanin, and other bioactive compounds such as ascorbic acid, flavonoids and hydroxycinnamic acid (Grosso et al., 2013). These compounds are associated with both antioxidant activity and cytoprotective effects that prevent chronic pathological conditions, certain cancers and cardiovascular disease (Butelli et al., 2012; Toker et al., 2014). Additionally, the blood orange has also attracted the attention of researchers for the application of these beneficial compounds for weight management and obesity in recent years (Butelli et al., 2012; Cardile et al., 2015). Although blood oranges have been the subject of extensive studies during the past decade, little is known about its cytogenetic characteristics such as karyotypes.

Most modern varieties of blood orange have been derived from old Italian varieties (Butelli et al., 2012), and currently, Sanguinello, Moro and Tarocco, represent the most common varieties of blood oranges (Caruso et al., 2016). The fruit of the Moro variety is the most strikingly red and has a sweet flavor with a hint of raspberry, which was documented to arise in the early 19th century near Lentini from the bud mutation of “Sanguinello Moscato.” The Sanguinello variety is also a “full-blood” orange with similar characteristics to the Moro variety (Grosso et al., 2013). Tarocco is the medium-sized seedless fruit and is the most flavorful among the three varieties (Caruso et al., 2016). The red pigmentation comes from retrotransposon-mediated transcriptional activation of the Ruby MYB-like transcription factor (Butelli et al., 2012). Seedlessness has become a prime breeding objective for all fresh citrus fruit according to the market and consumer’s preference (Ollitrault et al., 2008; Grosser and Gmitter, 2011). Triploid has become an important strategic tool in the development of commercial citrus varieties with the highly desirable trait of seedlessness. Triploids can be generated directly from artificial and spontaneous mutations and from hybridization between diploid and tetraploid parental genotypes (Grosser and Gmitter, 2011; Aleza et al., 2012).

In our previous study, we identified and obtained a naturally occurring tetraploid blood orange plant via seedling screening. Given the usefulness of a tetraploid for seedless triploid scion breeding programs, there is a need for a thorough investigation of its chromosomal characteristics and composition to elucidate its tetraploid formation pathway. Karyotypes, displaying chromosome number, morphology, and composition, are a basic cytological characterization of each species and has become a central issue in the study of plant cell biology, providing useful molecular cytogenetic information not only for plant taxonomy and phylogenetics studies but also for further breeding programs (Liang and Chen, 2015). A key prerequisite to establish karyotypes is the ability to distinguish individual chromosome pairs of the species (Jiang and Gill, 2006). However, the somatic metaphase chromosomes of all citrus species are rather small (2–4 μm) (Krug, 1943). In addition, morphologically similar chromosomes and the paucity of effective chromosome-specific markers make it difficult to accurately discriminate individual chromosome pairs during karyotype analyses that depend solely on conventional cytogenetic approaches in citrus.

In the past few decades, fluorescence *in situ* hybridization (FISH) targeting repetitive DNA sequences such as satellite repeats and ribosomal RNA (rDNA) gene repeats has greatly assisted in chromosome discrimination for karyotype analysis in several plants (Cai et al., 2014; He et al., 2015; Huang et al., 2016; Li et al., 2018). Repetitive DNA sequences compose a large fraction of higher plant genomes and may vary greatly between species (Heslop-Harrison and Schwarzacher, 2011; Garrido-Ramos, 2015, 2017). Satellite repeats (i.e., tandemly repeated sequences) can generate specific distribution patterns on chromosomes through FISH (Jiang and Gill, 2006; Cai et al., 2014; He et al., 2015; Huang et al., 2016), as some repeats are well conserved, while others are fast-evolving components of eukaryotic genomes (Heslop-Harrison and Schwarzacher, 2011; Garrido-Ramos, 2015, 2017). 45S rDNA and 5S rDNA are two types of rDNA gene repeats found in plants; they are expressed as essential housekeeping genes in eukaryotes, and the abundance of rDNA maintains genome integrity (Ide et al., 2010). 45S rDNA encoding 18S-5.8S-26S rRNA genes generally refer to the secondary constriction at the nucleolar organizing regions (NORs) (Heslop-Harrison and Schwarzacher, 2011; Garcia et al., 2017).

In our present work, we used four satellite repeats, a centromere-like repeat, two rDNAs and an oligonucleotide of (TTTAGGG)_3_ as chromosomal markers. Sequential multicolor FISH mapping with these chromosomal markers was conducted on the somatic metaphase chromosomes of a diploid blood orange and this naturally occurring tetraploid plant. Fine karyotypes were established via holistic chromosomal characteristics. The chromosome complement of the tetraploid was analyzed, and the possible pathway involving tetraploid formation was discussed. The present investigations can provide valuable molecular cytological characterization of blood orange and may serve as the starting point for the utilization of tetraploid germplasm in the service of future seedless breeding programs for citrus improvement.

## Materials and Methods

### Plant Materials

The mature leaves of Clementine mandarin (*C. clementina* Hort. ex Tan.) mature leaves for DNA extraction in this study were collected from the National Germplasm Repository, Citrus Research Institute, Chinese Academy of Agricultural Sciences, which is located in Chongqing, China (latitude 23.39°N, longitude 34.95°E). The diploid and tetraploid blood orange plants were obtained from a seedling screening in our laboratory and grown under natural field condition in the experimental block of College of Horticulture and Landscape Architecture, Southwest University, Chongqing, China. Fresh root tips harvested from the plants were used as the source of mitotic cells for chromosome preparation. The ploidy level of plants has been determined by somatic chromosome counting and flow cytometric measurement before sampling.

### DNA Target Preparation

Total genomic DNA was extracted from 100 mg fresh leaves of Clementine mandarin using the DNeasy^®^ Plant Mini kit (Qiagen; Valencia, CA, United States) according to the manufacturer’s instructions. Four tandem-arrayed DNA sequences (i.e., CL1, CL2, CL3, and CL4), a centromere-like repeat (CL17), and two rDNAs (45S and 5S rDNA) were isolated from Clementine genome^1^ through RepeatExplorer in Galaxy^2^ exactly as detailed in Deng et al. (2019). The Clementine genomic DNA was used as the template for PCR amplifications. The amplification of these repeats was carried out with the specific primers (Table 1) following Deng et al. (2019). PCR product was cloned into the pGEM^®^-T Easy vector and propagated in *Escherichia coli* DH5α^TM^ cells according to the manufacturer’s recommendations.

**Table 1:** Chromosomal localization of satellite DNA repeats and rDNA in diploid and tetraploid blood orange plants.

Probe name	Probe type	sites/2X	sites/4X	Localization by *in situ* hybridization	Primers for PCR amplification
CL1	Satellite DNA	13	26	Terminal region of q arm in chromosome pair No. 2, 3, 4, 5, and 6; Terminal region of p arm in chromosome pair No. 3; One weak signal located in p arm of only one chromosome No. 4.	F: CCGCAAAGTCTCGGGCCAT, R: CGCCCAAAAATTAGCGCCCGAAG
CL2	Satellite DNA	16	32	Terminal region of q arm in chromosome pair No. 2, 3, 4, 5, and 6; proximal regions of p arm in chromosome pair No. 2; Terminal region of p arm in chromosome pair No. 3; One weak signal located in p arm of only one chromosome No. 4.	F: TCGGAATGGCGCGAGACTTT, R: GGCCTTTTTCCGCTGGACGA
CL3	Satellite DNA	13	26	Terminal region of q arm in chromosome pair No. 2, 3, 4, 5, and 6; Terminal regions of p arm in chromosome pair No. 3; One weak signal located in p arm of only one chromosome No. 4.	F: CTGCGCGCGATGGTGCCTC, R: GCGCGAAACTAGCCCGCCAAC
CL4	Satellite DNA	12	24	Terminal region of q arm of chromosome pair No. 2, 3, 4, 5, and 7; Terminal region of short arm of chromosome pair No. 3.	F: TCATGCCCATTTTTCGGCGTTC, R: GGGCCTCCGATTCCGTTTCC
45SrDNA	Ribosomal RNA	3	6	Proximal regions of p arm of chromosome pair No. 2; Satellite region of q arm of chromosome pair No. 8	F: ACTAAGAACGGCCATGCACCA, R: ATCCTGCCAGTAGTCATAGCTT
5SrDNA	Ribosomal RNA	2	4	Terminal regions of q arm of chromosome pair No. 8	F: ACAATGTCTTCCGCCCGGATC, R: GGCCGAAGAGGGGAAAGGTTC
CL17	Centromere-like repeat	18	36	Centromere area of each chromosome pair	F:CTGTTTGTCCATCTTCAAGGGG, R: CTTTCTGTTGAGATGAGTGTCCG
TTTAGGG	Telomere repeat	36	72	Distal telomere region of all chromosome pairs	(TTTAGGG)3


### Nick-Translation Probe Labeling

An oligonucleotide of (TTTAGGG)_3_ was synthesized by Shanghai Invitrogen Biotechnology Co., Ltd., with digoxigenin labeled at the 5′ end. The plasmid was labeled with nick translation reaction following the protocol described by Cai et al. (2014). Briefly, the nick-translation probe labeling reaction solution contained 5 μL of 10X nick-translation buffer, 2.5 μl of dNTP solution, 2.5 μl of dUTP solution [either biotin-16-dUTP, digoxigenin-11-dUTP, CY5-dUTP (Roche, Basel, Switzerland), or diethylaminocoumarin-5-dUTP (PerkinElmer Inc., Boston, MA, United States)], 1 μg of the target plasmid DNA, and 12 units of nick-translation enzyme mixture of deoxyribonuclease I (DNase I) and DNA polymerase I, with a final reaction volume of 50 μl. 5 μL of stop buffer (0.5 M EDTA buffer, pH 8.0) was added to terminate nick translation after incubation at 15°C for 2.5 h. The size of the labeled DNA fragments was determined with 1% agarose gels. The majority of fragments ranging from 200–600 bp were considered suitable sizes for FISH probes.

### Metaphase Chromosome Preparation

Chromosome preparation and the FISH procedure were performed according to the previously reported procedure in our laboratory (Cai et al., 2014; He et al., 2015; Huang et al., 2016; Zhu et al., 2016) with a minor improvement. Briefly, root tips (approximately 1–2 cm long) were collected into a moist filter paper and quickly exposed to nitrous oxide at a 2 psi for 2 h. The treated root tips were then fixed in 3:1 (absolute ethanol: glacial acetic acid, v/v) Carnoy’s fixative solution for 2 days at room temperature (25°C) and then stored at -20°C until use. Subsequently, the fixed roots were washed with distilled water. Root apexes containing actively growing cells were macerated in an enzyme mixture containing 3% (w/v) cellulase Onozuka R-10 (Yakult Pharmaceutical Industry Co., Ltd., Tokyo, Japan) and 0.3% (w/v) pectolyase Y-23 (Kikkoman Corp., Tokyo, Japan) for 2 h at a 37°C water bath, and then the enzyme solution was replaced with deionized water. The digested root tips were gently placed on slides with two drops of 3:1 Carnoy’s fixative solution and immediately flame-dried. The slides with well-spread somatic metaphase chromosomes were screened under phase contrast optics.

### Sequential Multicolor FISH Procedure

The slides were denatured with 100 μl of 70% formamide in 2× SSC buffer for 90 sec in an 85°C hot oven, dehydrated in an increasing ethanol series for 5 min each (70, 90, and 100%) at -20°C, and air dried. A hybridization mixture containing 10 μl of deionized formamide, 2 μl of 20× SSC, 2 μl of sheared salmon sperm DNA (10 mg/ml), 4 μl of 50% dextran sulfate and 2.0 μl denatured probes was denatured at 90°C for 10 min in a metal bath in dark conditions, immediately chilled on ice for 10 min and briefly centrifuged before use. A 20 μl denatured hybridization mixture was added to the area containing the chromosome spreads and covered with an 18 × 18 mm coverslip. Hybridization was performed at 37°C for at least 8 h in a moist chamber. Posthybridization washes were done successively with 2× SSC and 1× PBS buffers for 5 min each. The biotin and digoxigenin labeled probes were detected by using anti-avidin antibody conjugated with FITC (Vector Laboratories, Inc., Burlingame, CA, United States) and anti-digoxigenin antibody coupled with Rhodamin (Roche), respectively. DEAC and CY5 labeled probes were detected directly. Chromosomes were mounted in a thin layer of VECTASHIELD^®^ Antifade Mounting Medium (Catalog No: H-1200, Vector Laboratories) containing 1.5 μg/ml 4′, 6-diamidino-2-phenylindole (DAPI). The *in situ* hybridization signals were captured by an Olympus BX61 epifluorescence microscope (Tokyo, Japan) equipped with a Sensys CCD camera (Qimaging Retiga^TM^ SRV Fast 1394, Vancouver, BC, Canada). FISH chromosome parameters and hybridization signals were measured by Image-Pro plus 6.5 software (Media Cybernetics). Images were adjusted with the aid of the Adobe Photoshop CS6.0 and ImageJ software (National Institutes of Health, Wayne Rasband, MD, United States). Before the consecutive round of FISH procedure, the slides were washed with a set of PBS (phosphate buffer saline) buffer and ethanol series to remove FISH hybridization signals from the previous round.

### Karyotype Analysis

Karyotype formulae were based on at least nine high-quality mitotic metaphase spreads. The degree of karyotype asymmetry was estimated with Stebbins’s method (Stebbins, 1971). Chromosome classifications were made by a standardized nomenclature proposed by Levan et al. (1964). According to this criterion of the centromeric position, chromosome homologs were identified as M, m, sm, st, t, and T having an arm ratio of 1.0, 1.01∼1.7, 1.71∼3.0, 3.01∼7.0, 7.01∼∞, and ∞, respectively. A cytogenetic tool, KaryoType software version 2.0, was used to account for the karyological parameters automatically (Altinordu et al., 2016). Statistical analysis was conducted using JMP Pro.14.1 software (SAS Institute Inc., Cary, NC, United States). Means and standard deviations were calculated. The lengths of the chromosomes in the ideograms were based on these mean values. Chromosomes were arranged in order of decreasing length to establish a fine karyotype analysis.

## Results

### Chromosomal Distribution Patterns of Satellite Repeats and rDNA in Diploid and Tetraploid Blood Orange Plants

**FIGURE 1:**
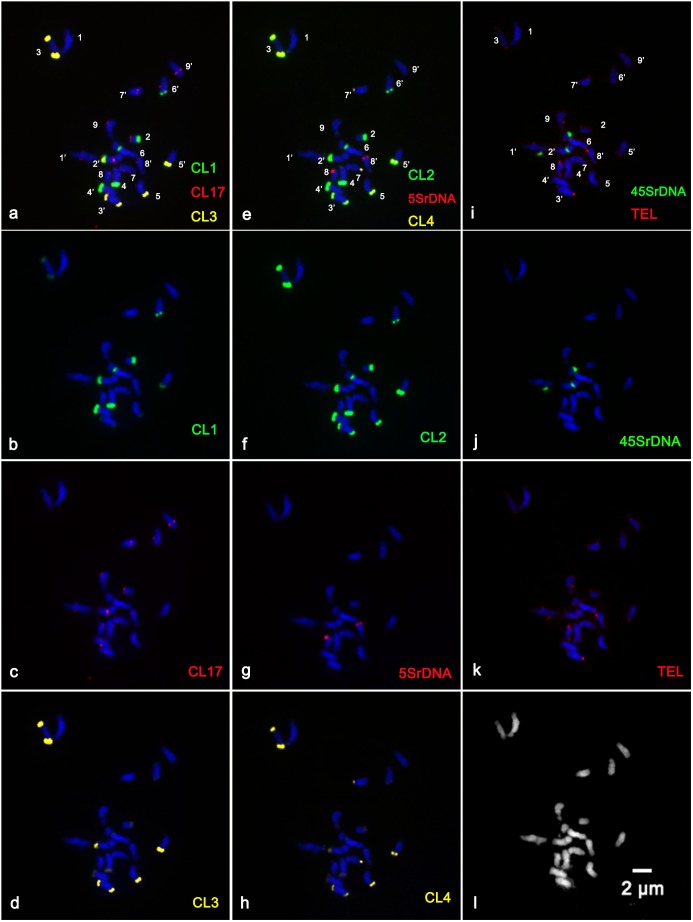
Distribution of the repetitive DNA probes on the somatic metaphase chromosomes of diploid blood orange using sequential multicolor FISH. The mitotic metaphase chromosomes came from the same metaphase spreads. Chromosomes were counterstained with DAPI (blue color). **(b)** Satellite repeat CL1 (green); **(c)** a centromere-like repeat CL17 (red); **(d)** satellite repeat CL3 (yellow); **(f)** satellite repeat CL2 (green); **(g)** 5S rDNA (red); **(h)** satellite repeat CL4 (yellow); **(j)** 45S rDNA (green); **(k)** telomere repeat (red); and **(l)** the morphology of the unstained chromosomes. The hybridization sites in **(b–d)**, **(f–h)**, and **(j–k)** were digitally separated from the merged images of **(a,e,i)**, respectively.

**FIGURE 2:**
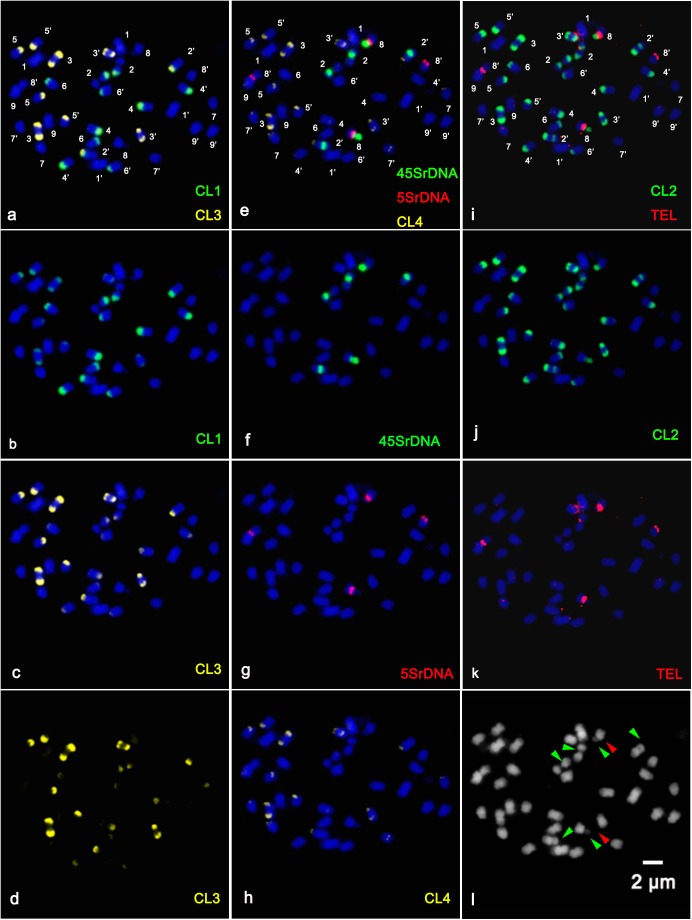
Distribution of the repetitive DNA probes on the somatic metaphase chromosomes of tetraploid blood orange using sequential multicolor FISH. The mitotic metaphase chromosomes came from the same metaphase spreads. Chromosomes were counterstained with DAPI (blue color). **(b)** Satellite repeat CL1 (green); **(c)** satellite repeat CL3; **(d)** separate signals of CL3 (yellow); **(f)** 45S rDNA (green); **(g)** 5S rDNA (red); **(h)** satellite repeat CL4 (yellow); **(j)** satellite repeat CL2 (green); **(k)** telomere repeat (red); and **(l)** the morphology of the unstained chromosomes. The hybridization sites in **(b–d)**, **(f–h)**, and **(j–k)** were digitally separated from the merged images of **(a,e,i)**, respectively. The red arrowhead shows the satellite region of the chromosome, and the green arrowhead shows the fragile sites of 45SrDNA.

**FIGURE 3:**
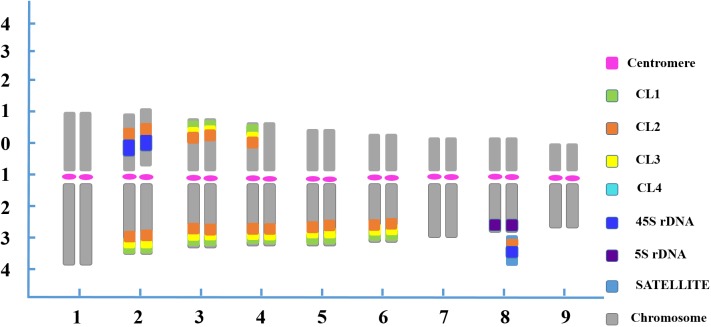
FISH-based ideogram of blood orange somatic metaphase chromosome. The short arm is positioned on top by convention. Numbers on the *x*-axis (1–9) indicate the pairs of homologous chromosomes. Numbers on the *y*-axis represent the relative chromosome length based on the mean morphometric parameters from Supplementary Table S1. Chromosome discriminations are based on not only on the distribution of the multicolor FISH signals (Table 1) but also the averaged morphometric parameters (Supplementary Table S1).

In the present study, sequential multicolor FISH on mitotic metaphase chromosomes showed that the CL1 probe generated 13 signals, of which ten were hybridized at the terminal position of the long arms of chromosome pairs 2, 3, 4, 5, and 6, two were located at the ends of the short arms of chromosome pair 3, and only one faint hybridization site was located at the terminal position of the short arm of one homolog of chromosome pair 4 (Figure 1a,b and Supplementary Figures S1a,b,e). The CL3 probe presented similar hybridization patterns to those of CL1 regarding number and localization, but they varied substantially in the size and intensity of the FISH signals (Figure 1a,d and Supplementary Figures S1a,c,f). The CL2 signals were detected at the terminal region of the long arms of chromosome pairs 2, 3, 4, 5, and 6, the pericentromeric region of the short arms of chromosome pair 2, the terminal position of the short arms of chromosome pair 3, and the terminal position of only one short arm of chromosome pair 4 and only one long arm of chromosome pair 8 (Figure 1e,f). The CL4 signals appeared at the terminal region of the long arm of chromosome pairs 2, 3, 4, 5, 7 and the terminal position of the short arm of chromosome pair 3 (Figure 1h, 3). FISH with a CL17 probe consistently hybridized at the centromeric positions of all chromosomes (Figure 1a,c). The telomere repeat (TTTAGGG) showed reproducible FISH hybridization signals on the distal telomere region of all chromosome pairs (Figure 1i,k). There were two 45S rDNA fragile sites hybridized on NORs (nuclear organization region) of chromosome pair 2 (Figure 1i,j). Two signals of 5S rDNA sites (Figure 1e,g) were clearly detected on chromosome pair 8. One of the 5S rDNA sites was terminally located on the long arm, and the other was colocalized with the third 45S rDNA site on the satellite region of the long arm of chromosome pair 8. The visualized linked site of 45S and 5S rDNA covered approximately all the satellite regions (Figure 1g,j and Supplementary Figure S2). Regarding chromosomal localizations, the naturally occurring tetraploid plant had a distribution pattern similar to that of the diploid plant, but the former carried twice the hybridization loci as the latter (Figure 2). The chromosomal localizations of satellite DNA and rDNA in diploid and tetraploid blood orange plants are summarized in Table 1.

### Karyotype Features of Diploid and Tetraploid Blood Orange Plants

All of the investigated diploid individuals had an invariable chromosome number of 2*n* = 18, and the tetraploid individuals had 2*n* = 36 chromosomes. The somatic metaphase chromosomes were relatively small in size and morphologically similar under a microscope (Figure 1l, 2l). The karyotype was formulated as 2*n* = 2*x* = 18 = 16m + 2sm (1sat). According to the classification system of Stebbins, the karyotype asymmetry degree belongs to 2B. The karyotype formulae of tetraploid are 32m + 4sm (2sat) with their complements mainly composed of metacentric (m) chromosomes, with two satellite chromosomes. Total haploid length of the chromosome set (THL) was 136.61 ± 3.87 μm. Other karyotype parameters including coefficient of variation of centromeric index, coefficient of variation of chromosome length, and mean centromeric asymmetry are given in Supplementary Table S1.

The physical mapping of a combination of four satellite DNA repeats, the two rRNA gene families 5S and 18S-5.8S-26S (45S), a centromere-like repeat and a telomere repeat by successive rounds of multicolor FISH was first applied to analyze in detail the karyotype characterization of the blood orange plants in this study. The reproducible multicolor FISH signals (Figure 1, 2) and morphometric parameters (Supplementary Table S1) enabled the identification of individual homologous chromosome pairs in both the diploid and tetraploid chromosome complements in the present study. From these observations, we delineated an ideogram representing the somatic karyotype of blood orange that included these repetitive DNA sequence locations.

In the karyotype, chromosome pair 1 was characterized by the largest size among all the homologous chromosome pairs. Chromosome pair 2 was easily recognized by two strong 45S rDNA loci located at the NOR region of the short arm adjacent to the CL2 locus, and four pairs of satellite DNA repeat loci at the ends of the long arms. Chromosome 3 exhibited robust satellite repeat loci on the distal position of both the long and short arms. One chromosome 4 had CL1, CL2 and CL3 loci on both the short and long arms, whereas its homolog had those signals only on the long arm. Chromosome 4 also carried two CL4 loci adjacent to the other satellite repeats located on the terminal position of the long arm. Chromosome pairs 5 and 6 were morphologically similar, but the former bearing brighter CL3 FISH signals was discernible from the latter bearing brighter CL1 FISH signals in symmetry at the terminal zone of the long arms. A characteristic feature of chromosome 7 was a pair of CL4 loci that was located terminally on the long arm. Chromosome 8 was instantly distinguished from chromosome complement with 5S rDNA loci in the terminal part of the long arms. Additionally, one 45S rDNA locus and one CL2 locus were simultaneously visualized on one chromosome 8 at the satellite region, representing the secondary constriction of the NOR. Despite the scarcity of special markers on chromosome pair 9, the pair was distinguishable as it had the smallest morphology and size (Figure 3).

The chromosomal features of this naturally occurring tetraploid blood orange plant were also thoroughly analyzed. All the tetraploid metaphase spreads displayed doubled FISH signals when compared to the diploid but with the same and identical distribution patterns, demonstrating that the distribution pattern is relative to the duplication of chromosomes in nucellar cells (Figure 3). Similar chromosomal characteristics could be identified for the diploid and tetraploid plants in our study; therefore, only one ideogram is presented (Figure 3). Taken together, sequential multicolor FISH-based karyotypes were established by using four tandem repeats in combination with two rDNAs, a centromere-like repeat and a telomere repeat, which enabled unambiguous and reproducible chromosome discrimination.

## Discussion

### The Improved Karyotype of Blood Orange

Fluorescence *in situ* hybridization allows the physical mapping of targeted genetic markers directly onto cytological chromosome preparations, thus providing reliable markers for accurate identification of individual chromosomes and karyotype analyses (Jiang and Gill, 2006). FISH-based karyotyping analysis can create the basis for the integration of molecular, genetic and cytological maps of plants (Jiang and Gill, 2006; Cai et al., 2014; He et al., 2015; Huang et al., 2016; Zhu et al., 2016). Regarding molecular cytogenetic studies, blood orange are a neglected fruit, although they are economically important sources of fresh fruits and there have been substantial other studies performed as described in the introduction section.

Blood orange chromosomes are rather small as the length of the chromosomes of *Citrus* L. varied between 2 and 4 μm (Krug, 1943), which results in intrinsic limitations for advances in traditional karyotype descriptions. In the present study, the karyotype formulation of blood orange suggests that the chromosomes of blood oranges are morphometrically indistinguishable, as most of which are metacentric to submetacentric, 2*n* = 2*x* = 18 = 16m + 2sm (1sat). Hence, unambiguous and reproducible chromosome discrimination has not been readily possible so far by traditional karyotyping without chromosome-specific markers, particularly taking into consideration the differences in chromosome condensation rates and arms bending up or down in the chromosome preparations (Figure 1a,g,h, 2a,d,h).

A previous karyotyping based on the length of mitotic metaphase chromosomes and 45S rDNA markers could not unambiguously discriminate all individual chromosome pairs (Lu et al., 2010). In that article, three 45S rDNA loci were detected in blood orange, two of which were localized on the short arms, close to the centromere constriction of chromosome 2, and another 45S rDNA locus was localized to the proximate telomere on the long arm of one chromosome 7; however, the karyotyping analysis was restricted to the 45S rDNA gene and a *dfr* gene (named CsDFR-bo) (Lu et al., 2010). FISH with chromosome-specific probes allows the clear recognition of individual chromosome pairs and their complements (Jiang and Gill, 2006; Cai et al., 2014; He et al., 2015; Huang et al., 2016; Zhu et al., 2016). In this study, we performed multicolor FISH-based karyotyping with consecutively applied sets of probes consisting of four major satellite repeats, a centromere-like repeat, two ribosomal genes and an oligonucleotide of a telomere repeat. Our results partially supported Lu et al.’s conclusion regarding the site numbers of 45S rDNA.

Additionally, to our knowledge, the blood orange is a natural mutation of the sweet orange (*C. sinensis*), which is generally perceived to have heterozygosity in several previous molecular studies (Nicolosi et al., 2000; Xu et al., 2013). Here, the asymmetric and odd FISH signals on chromosome pairs 4 and 8 confirmed the hybrid origin of the blood orange (Figure 3). The heterozygosity in blood orange was cytogenetically confirmed in our study. Conclusively, by applying repetitive DNA-based multicolor FISH to blood orange somatic metaphase chromosomes, most of the homologous chromosome pairs were characterized by one or more markers, resulting in significantly increased chromosome specificity. This provided unambiguous and reproducible chromosome identification to develop a greatly improved FISH-based karyotype of blood orange. Karyotype features represent important aspects of cytogenetic studies (Liang and Chen, 2015). Accordingly, this karyotyping system might be a starting point for more advanced studies of the genomics, phylogenetics and taxonomy of blood orange in the future.

### Good Chromosomal Landmark-Repetitive DNA Sequences

Here, the chromosomal distribution patterns of the repetitive DNAs greatly facilitated chromosome discrimination and developed an accurate and reliable karyotype. Repetitive DNA sequences are the major contributors to the chromosomal structure in plants (Heslop-Harrison and Schwarzacher, 2011). Variation in sequence, repeat monomer length, copy number, abundance and chromosome location of repetitive DNAs found among individuals and species directly affect genomic organization and evolutionary dynamics (Biscotti et al., 2015). Therefore, it has been well documented for a number of plant species that the identification of individual chromosomes with repetitive DNAs as markers is a very powerful and effective methodology (Cai et al., 2014; He et al., 2015; Zhu et al., 2016).

Satellite repeats are fast-evolving elements of most multicellular eukaryotic genomes, usually associated with condensed heterochromatin and arranged as arrays of highly abundant, head-to-tail tandem repetitions (Biscotti et al., 2015; Garrido-Ramos, 2015). Satellite arrays are usually located in the heterochromatin and may contribute to essential chromosomal structures such as centromeric and telomeric regions (Heslop-Harrison and Schwarzacher, 2011; Biscotti et al., 2015; Garrido-Ramos, 2017). Chromosomal localization of the satellite DNA repeats was mainly in the telomeric areas of chromosomes but was also in the pericentromeric area of chromosome pair 2 (Figure 1, 2). In the present work, the preferential localization of satellite repeats in the terminal and subterminal regions of blood oranges observed in our study was a common and typical feature of satellite DNA repeats (Table 1; Heslop-Harrison and Schwarzacher, 2011; Biscotti et al., 2015; Garrido-Ramos, 2017). According to previous reports of satellite repeats and heterochromatin in *Citrus* L., the heterochromatin-associated markers showed a preferential accumulation partially overlapping with the CMA^+^ heterochromatin (Marques et al., 2011); the satellite repeats were the principal component of heterochromatic blocks of *Citrus* and its related genus (Silva et al., 2010). Thus, the chromosomal localization of the satellite repeats was in agreement with the previous results.

Sites of rDNA are by far the most extensively reported chromosomal regions (Heslop-Harrison and Schwarzacher, 2011). Here two hybridized signals were detected in the NOR regions of homologous chromosome pair 2 and almost the whole satellite region of one homologous chromosome pair 8 (Figure 1i, 2f and Supplementary Figure S2). In a recent experiment relating to the 45S rDNA sites in sweet orange (*C. sinensis*), Lan et al. (2016) proposed that a close connection exists between 45S rDNA sites and fragile sites. This information would be of great assistance in the interpretation of characteristics of chromosome breakages or gaps in the homologous chromosome pair 2 observed in this study (Figure 3 and Supplementary Figure S1). Regarding other existing representative studies on rDNA distribution in *Citrus*, Silva et al. summarized that the linked 5S and 45S rDNA sites are highly conserved through the subfamily Aurantioideae (Rataceae) (Silva et al., 2013). In addition the chromosomal localization of CL17 made it a good candidate marker for chromosome identification by determining the centromeric position (Figure 1c). The oligonucleotides of (TTTAGGG)_3_ signals were detected at telomeric positions on both the short and long arms of the chromosomes (Figure 1i), indicating that there might be high homology and conservation of telomere DNA between different chromosomes. We can conclude that the simultaneous localization of these repetitive DNAs indicated the specific chromosomal landmarks and enabled more precise information about chromosome morphology and structure.

### Tetraploidization in a Naturally Occurring Blood Orange Plant by the Chromosome Doubling of Nucellar Cells

Polyploidy or whole-genome duplication events, followed by gene loss and diploidization, have been widely considered a major force in the plant evolutionary process (Sattler et al., 2016; Van de Peer et al., 2017). Generally, polyploid organisms are often more vigorous than their diploid relatives in several aspects (Sattler et al., 2016).

In citrus germplasm, diploidy (2*n* = 2*x* = 18) is the prevailing state (Krug, 1943; Gmitter et al., 2012). During recent years, polyploids such as triploid, tetraploid, pentaploid (Aleza et al., 2012), hexaploid (Aleza et al., 2012), heptaploid (Pensabene-Bellavia et al., 2015), octaploid (Usman et al., 2006), and aneuploidy citrus have been identified or obtained spontaneously or artificially. The most extensively studied euploid variations are triploids and tetraploids (Lee, 1988). Polyploidy in citrus was studied as early as 1925 when Longley formally identified a tetraploid wild “Hong Kong” kumquat (*Fortunella hindsii* Swing.) (Longley, 1925). Triploid “Tahiti” lime (*C. latifolia* Tan.), tetraploid strains of *Poncirus trifoliata* (L.) Raf., allotetraploid *Clausena excavate* Burm.F., tetraploid *Clausena harmandiana* Pierre (Guill) and hexaploid *Glycosmis pentaphylla* Retz. (Correa) are other examples of naturally occurring polyploids in the germplasm of Aurantioideae (Ollitrault et al., 2008; Gmitter et al., 2012). The possible predominant pathway leading to polyploidy in plants is somatic doubling and the formation of unreduced reproductive cells (Sattler et al., 2016). Polyploid citrus can be obtained via bud sports, the establishment of interspecific somatic hybridization, somatic cell fusion, embryo rescue, endosperm culture and mutation breeding either in the context of citrus breeding or in wild populations (Ollitrault et al., 2008; Grosser and Gmitter, 2011).

As research in the field of tetraploidization event in citrus has progressed, Barrett has proposed a tetraploid frequency of less than 1–3% depending on genotype (Barrett, 1977). In a more recent experiment, tetraploidization events by chromosome doubling of nucellar cells have also been reported to be frequent events (Aleza et al., 2011). The variable frequency of spontaneous polyploid citrus plants relying on genetic and environmental control can be seen in the paper by Hussain (Hussain et al., 2012). In a similar investigation, Guerra et al. (2016) showed that the frequency of autotetraploidization events in citrus rootstocks is common, and his work has shed light on the tetraploidization events in relation to genetic constitution and environmental conditions.

There are two main hypothesized mechanisms that could lead to this naturally occurring tetraploid blood orange plant in this study (Sattler et al., 2016; Van de Peer et al., 2017). A plausible explanation is that the tetraploid formation was highly relevant in the chromosome doubling of nucellus cells. Another possibility is that 2n gametes participated in the formation of tetraploids. We found that the tetraploid metaphase chromosomes displayed similar distribution patterns to those of the diploid blood orange plant, but carried twice the number of sites as the diploid (Figure 1, 2, and Table 1). Cytogenetically speaking, this naturally occurring tetraploid blood orange plant arose from the duplication of chromosomes in nucellar cells and the lack of division during mitosis. This would be a scenario analogous to previous findings that those “spontaneous tetraploids” with identical molecular profiles to their respective diploid parent plants are the results of duplication of the chromosome set of nucellar cells (Aleza et al., 2011; Hussain et al., 2012; Guerra et al., 2016). Furthermore, it is intriguing that the size of the homologous chromosome pairs were not always identical, such as chromosome pair 2 (Figure 3 and Supplementary Figures S1, S2). This phenomenon observed in the present study was likely indicative of a few chromosome breakage and translocation events during the evolutionary process. These chromosomal variants would be a very interesting topic to study in the future. As discussed above, tetraploid germplasms are often of considerable interest in breeding triploid seedlessness. Our study helped to characterize this naturally occurring tetraploid germplasm at the cytological level well before the germplasms can be effectively manipulated.

## Conclusion

In this article, a set of repetitive DNA probes was used for sequential multicolor FISH to establish the cytogenetic karyotype of blood orange. The probes contained four satellite repeats, two rDNAs, a centromere-like repeat and an oligonucleotide of a telomere repeat of (TTTAGGG)_3_. Physical mapping of the repetitive DNAs loci by multicolor FISH enabled the unambiguous and reproducible identification of individual chromosome pairs of blood orange. Accordingly, these probes are considered good chromosomal landmarks. We built an integrated karyotype of blood orange. The naturally occurring tetraploid was cytogenetically confirmed to arise from the duplication of chromosomes in nucellar cells due to bearing the identical distribution pattern and twice the hybridization sites of satellite DNA repeats and rDNA as the diploid. Our work may facilitate molecular cytogenetic studies of blood orange and provide chromosomal characterization for the future utilization of this tetraploid blood orange in the service of seedless breeding programs.

## Author Contributions

GL and WH conceived and designed the research and revised the manuscript. HD performed the experiments and wrote and revised the manuscript. ZC helped with the karyotyping analysis. SX managed the material collection. SX and QG helped with the data analysis. All authors read and approved the final manuscript.

## Conflict of Interest Statement

The authors declare that the research was conducted in the absence of any commercial or financial relationships that could be construed as a potential conflict of interest.

## Footnotes

1 https://www.citrusgenomedb.org/species/clementina

2 http://repeatexplorer.org/

## Supplementary Material

FIGURE S1 Distribution of CL1 and CL3 on the somatic metaphase chromosomes of blood orange using sequential multicolor FISH. CL1 **(b,e)** and CL3 **(c,f)** showed the same site numbers and localization but varied in FISH intensities. **(a)** The merged image from **(b,c)**; and **(d)** the morphology of the unstained chromosomes. The arrow indicates one faint signal on the terminal position of the short arm of one homolog of chromosome pair 4.

FIGURE S2 Distribution of 45S and 5S rDNA on the somatic metaphase chromosomes of blood orange using multicolor FISH. The green arrowheads indicate the 45S rDNA loci (green signals), and the red arrowheads indicate the 5S rDNA loci (red signals).

TABLE S1 Karyotype parameters of blood orange.

## References

[B1] Aleza P. Froelicher Y. Schwarz S. Agusti M. Hernandez M. Juarez J. (2011). Tetraploidization events by chromosome doubling of nucellar cells are frequent in apomictic citrus and are dependent on genotype and environment. *Ann. Bot.* 108 37–50. 10.1093/aob/mcr099 21586529 PMC3119611

[B2] Aleza P. Juárez J. Hernandez M. Ollitrault P. Navarro L. (2012). Implementation of extensive citrus triploid breeding programs based on 4x × 2x sexual hybridisations. *Tree Genet. Genomes* 8 1293–1306. 10.1007/s00299-010-0888-7 20607244

[B3] Altinordu F. Peruzzi L. Yu Y. He X. (2016). A tool for the analysis of chromosomes: KaryoType. *Taxon* 65 586–592. 10.12705/653.9

[B4] Barrett H. (1977). Intergeneric hybridization of *Citrus* and other genera in citrus cultivar improvement. *Proc. Intl. Soc. Citricult.* 2 586–589.

[B5] Biscotti M. A. Olmo E. Heslop-Harrison J. S. (2015). Repetitive DNA in eukaryotic genomes. *Chromosome Res.* 23 415–420. 10.1007/s10577-015-9499-z 26514350

[B6] Butelli E. Licciardello C. Zhang Y. Liu J. Mackay S. Bailey P. (2012). Retrotransposons control fruit-specific, cold-dependent accumulation of anthocyanins in blood oranges. *Plant Cell* 24 1242–1255. 10.1105/tpc.111.095232 22427337 PMC3336134

[B7] Cai Z. X. Liu H. J. He Q. Y. Pu M. W. Chen J. Lai J. S. (2014). Differential genome evolution and speciation of Coix lacryma-jobi L. and Coix aquatica Roxb. hybrid guangxi revealed by repetitive sequence analysis and fine karyotyping. *BMC Genomics* 15:1025. 10.1186/1471-2164-15-1025 25425126 PMC4256728

[B8] Cardile V. Graziano A. C. Venditti A. (2015). Clinical evaluation of Moro (*Citrus sinensis* (L.) Osbeck) orange juice supplementation for the weight management. *Nat. Prod. Res.* 29 2256–2260. 10.1080/14786419.2014.1000897 25588369

[B9] Caruso M. Ferlito F. Licciardello C. Allegra M. Strano M. C. Di Silvestro S. (2016). Pomological diversity of the Italian blood orange germplasm. *Sci. Hortic.* 213 331–339. 10.1016/j.scienta.2016.10.044

[B10] Deng H. Xiang S. Guo Q. Jin W. Cai Z. Liang G. (2019). Molecular cytogenetic analysis of genome-specific repetitive elements in Citrus clementina Hort. Ex Tan. and its taxonomic implications. *BMC Plant Biol.* 19:77. 10.1186/s12870-019-1676-3 30770721 PMC6377768

[B11] Garcia S. Kovarik A. Leitch A. R. Garnatje T. (2017). Cytogenetic features of rRNA genes across land plants: analysis of the plant rDNA database. *Plant J.* 89 1020–1030. 10.1111/tpj.13442 27943584

[B12] Garrido-Ramos M. (2017). Satellite DNA: an evolving topic. *Genes* 8:230. 10.3390/genes8090230 28926993 PMC5615363

[B13] Garrido-Ramos M. A. (2015). Satellite DNA in plants: more than just rubbish. *Cytogenet. Genome Res.* 146 153–170. 10.1159/000437008 26202574

[B14] Gmitter F. G. Chen C. Machado M. A. de Souza A. A. Ollitrault P. Froehlicher Y. (2012). Citrus genomics. *Tree Genet. Genomes* 8 611–626. 10.1007/s11295-012-0499-2

[B15] Grosser J. W. Gmitter F. G. (2011). Protoplast fusion for production of tetraploids and triploids: applications for scion and rootstock breeding in citrus. *Plant Cell Tiss. Org.* 104 343–357. 10.1007/s11240-010-9823-4

[B16] Grosso G. Galvano F. Mistretta A. Marventano S. Nolfo F. Calabrese G. (2013). Red orange: experimental models and epidemiological evidence of its benefits on human health. *Oxid. Med. Cell Longev.* 2013:157240. 10.1155/2013/157240 23738032 PMC3659473

[B17] Guerra D. Schifino-Wittmann M. T. Schwarz S. F. Weiler R. L. Dahmer N. Souza P. V. D. D. (2016). Tetraploidization in citrus rootstocks: effect of genetic constitution and environment in chromosome duplication. *Crop Breed. Appl. Biol.* 16 35–41. 10.1590/1984-70332016v16n1a6

[B18] He Q. Y. Cai Z. X. Hu T. H. Liu H. J. Bao C. L. Mao W. H. (2015). Repetitive sequence analysis and karyotyping reveals centromere-associated DNA sequences in radish (*Raphanus sativus* L.). *BMC Plant Biol.* 15:105. 10.1186/s12870-015-0480-y 25928652 PMC4417506

[B19] Heslop-Harrison J. S. Schwarzacher T. (2011). Organisation of the plant genome in chromosomes. *Plant J.* 66 18–33. 10.1111/j.1365-313X.2011.04544.x 21443620

[B20] Huang W. Du Y. Zhao X. Jin W. (2016). B chromosome contains active genes and impacts the transcription of a chromosomes in maize (*Zea mays* L.). *BMC Plant Biol.* 16:88. 10.1186/s12870-016-0775-7 27083560 PMC4833949

[B21] Hussain S. Curk F. Dhuique-Mayer C. Urban L. Ollitrault P. Luro F. (2012). Autotetraploid trifoliate orange (*Poncirus trifoliata*) rootstocks do not impact clementine quality but reduce fruit yields and highly modify rootstock/scion physiology. *Sci. Hortic.* 134 100–107. 10.1016/j.scienta.2011.11.008

[B22] Ide S. Miyazaki T. Maki H. Kobayashi T. (2010). Abundance of ribosomal RNA gene copies maintains genome integrity. *Science* 327 693–696. 10.1126/science.1179044 20133573

[B23] Jiang J. Gill B. S. (2006). Current status and the future of fluorescence *in situ* hybridization (FISH) in plant genome research. *Genome* 49 1057–1068. 10.1139/g06-076 17110986

[B24] Krug C. A. (1943). Chromosome numbers in the subfamily aurantioideae with special reference to the genus citrus. *Botanical Gazette* 104 602–611. 10.1086/335173

[B25] Lan H. Chen C.-L. Miao Y. Yu C.-X. Guo W.-W. Xu Q. (2016). Fragile sites of ‘Valencia’sweet orange (*Citrus sinensis*) chromosomes are related with cctive 45s rDNA. *PLoS One* 11:e0151512. 10.1371/journal.pone.0151512 26977938 PMC4792391

[B26] Lee L. S. (1988). Citrus polyploidy-origins and potential for cultivar improvement. *Aust. J. Agric. Res.* 39 735–747. 10.1071/AR9880735

[B27] Levan A. Fredga K. Sandberg A. A. (1964). Nomenclature for centromeric position on chromosomes. *Hereditas* 52 201–220. 10.1111/j.1601-5223.1964.tb01953.x

[B28] Li D. Li T. Wu Y. Zhang X. Zhu W. Wang Y. (2018). FISH-based markers enable identification of chromosomes derived from tetraploid Thinopyrum elongatum in hybrid lines. *Front. Plant Sci.* 9:526. 10.3389/fpls.2018.00526 29765383 PMC5938340

[B29] Liang G. Chen H. (2015). Scaling chromosomes for an evolutionary karyotype: a chromosomal tradeoff between size and number across woody Species. *PLoS One* 10:e0144669. 10.1371/journal.pone.0144669 26657837 PMC4684206

[B30] Longley A. (1925). Polycary, polyspory, and polyploidy in *Citrus* and *Citrus* relatives. *J. Wash. Acad. Sci.* 15 347–351.

[B31] Lu X. Zhou W. Gao F. (2010). Chromosomal location of 45S rDNA and dfr gene in *Citrus sinensis*. *Biologia Plantarum* 54 798–800. 10.1007/s10535-010-0146-4

[B32] Marques A. Fuchs J. Ma L. Heckmann S. Guerra M. Houben A. (2011). Characterization of Eu- and heterochromatin of citrus with a focus on the condensation behavior of 45S rDNA chromatin. *Cytogenet. Genome Res.* 134 72–82. 10.1159/000323971 21304248

[B33] Nicolosi E. Deng Z. N. Gentile A. La Malfa S. Continella G. Tribulato E. (2000). Citrus phylogeny and genetic origin of important species as investigated by molecular markers. *Theor. Appl. Genet.* 100 1155–1166. 10.1007/s001220051419

[B34] Ollitrault P. Dambier D. Luro F. Froelicher Y. (2008). Ploidy manipulation for breeding seedless triploid citrus. *Plant Breed. Rev.* 30 323–352. 10.1002/9780470380130.ch7 19834711

[B35] Pensabene-Bellavia G. Ruiz M. Aleza P. Olivares-Fuster O. Ollitrault P. Navarro L. (2015). Chromosome instability in Carrizo citrange + Citrus macrophylla somatic hybrids. *Sci. Hortic.* 1065 677–685. 10.17660/ActaHortic.2015.1065.85

[B36] Sattler M. C. Carvalho C. R. Clarindo W. R. (2016). The polyploidy and its key role in plant breeding. *Planta* 243 281–296. 10.1007/s00425-015-2450-x 26715561

[B37] Silva A. E. B. E. Marques A. dos Santos K. G. B. Guerra M. (2010). The evolution of CMA bands in *Citrus* and related genera. *Chromosome Res.* 18 503–514. 10.1007/s10577-010-9130-2 20490650

[B38] Silva A. E. B. E. Soares W. D. Guerra M. (2013). Linked 5S and 45S rDNA sites are highly conserved through the subfamily aurantioideae (Rutaceae). *Cytogenet. Genome Res.* 140 62–69. 10.1159/000350695 23635472

[B39] Stebbins G. L. (1971). *Chromosomal Evolution in Higher Plants.* London: Arnold.

[B40] Toker R. Karhan M. Tetik N. Turhan I. Oziyci H. R. (2014). Effect of ultrafiltration and concentration processes on the physical and chemical composition of blood orange juice. *J. Food Process. Preserv.* 38 1321–1329. 10.1111/jfpp.12093

[B41] Usman M. Saeed T. Khan M. Fatima B. (2006). Occurrence of spontaneous polyploids in *Citrus*. *Hort. Sci.* 33 124–129. 10.17221/3751-HORTSCI

[B42] Van de Peer Y. Mizrachi E. Marchal K. (2017). The evolutionary significance of polyploidy. *Nat. Rev. Genet.* 18 411–424. 10.1038/nrg.2017.26 28502977

[B43] Xu Q. Chen L. L. Ruan X. A. Chen D. J. Zhu A. D. Chen C. L. (2013). The draft genome of sweet orange (*Citrus sinensis*). *Nat. Genet.* 45 59–66. 10.1038/ng.2472 23179022

[B44] Zhu Q. Cai Z. Tang Q. Jin W. (2016). Repetitive sequence analysis and karyotyping reveal different genome evolution and speciation of diploid and tetraploid *Tripsacum dactyloides*. *Crop J.* 4 247–255. 10.1016/j.cj.2016.04.003

