# Supplementary figures and images for: Karyotype Analysis of Diploid and Spontaneously Occurring Tetraploid Blood Orange [Citrus sinensis (L.) Osbeck] Using Multicolor FISH With Repetitive DNA Sequences as Probes
Source: Front Plant Sci. 2019 Mar 22;10:331. doi: 10.3389/fpls.2019.00331 (PMC6440391; doi:10.3389/fpls.2019.00331)

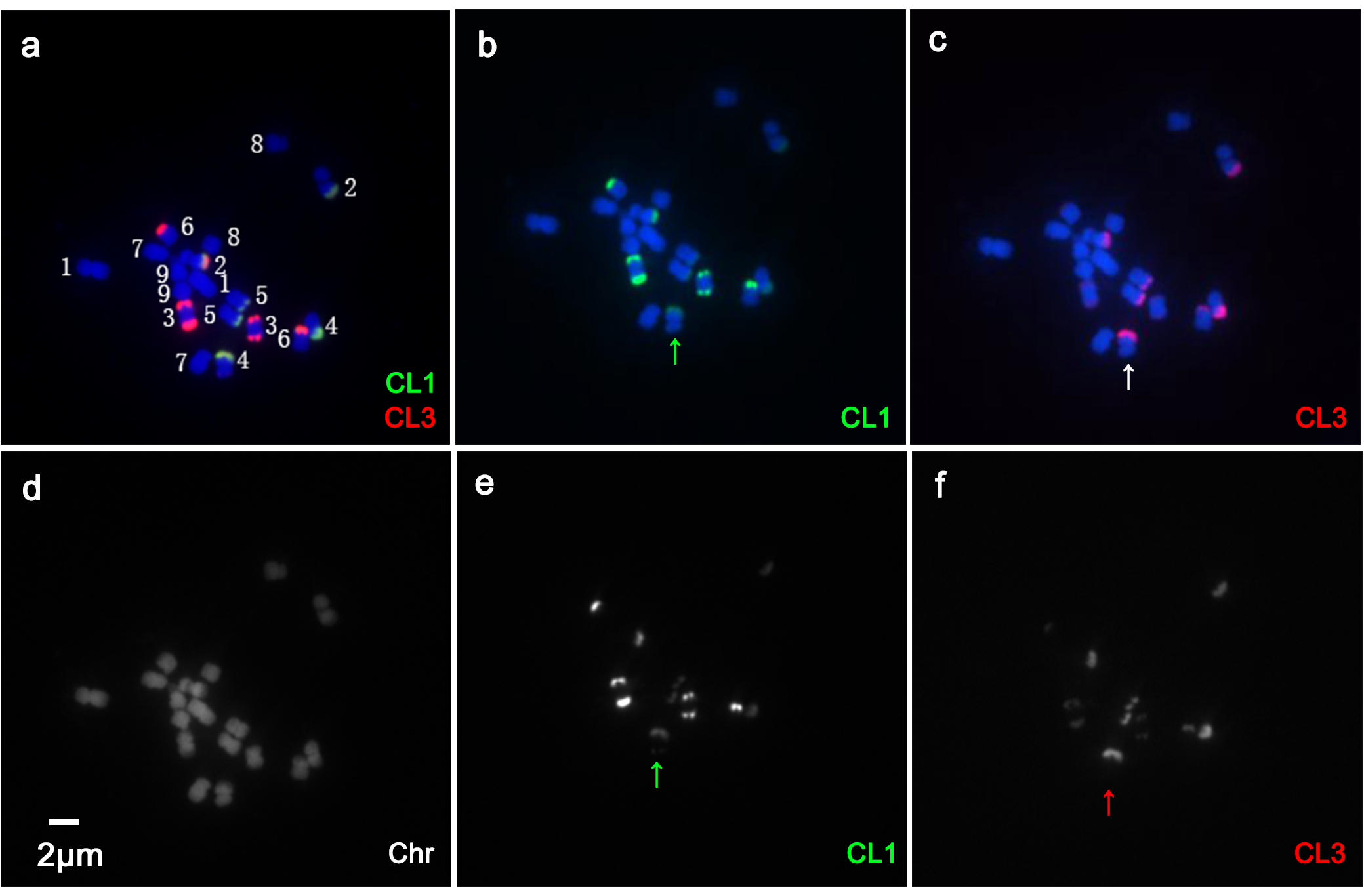

Supplement: FIGURE S1 — Distribution of CL1 and CL3 on the somatic metaphase chromosomes of blood orange using sequential multicolor FISH. CL1 (b,e) and CL3 (c,f) showed the same site numbers and localization but varied in FISH intensities. (a) The merged image from (b,c); and (d) the morphology of the unstained chromosomes. The arrow indicates one faint signal on the terminal position of the short arm of one homolog of chromosome pair 4. [file Image_1.TIF]

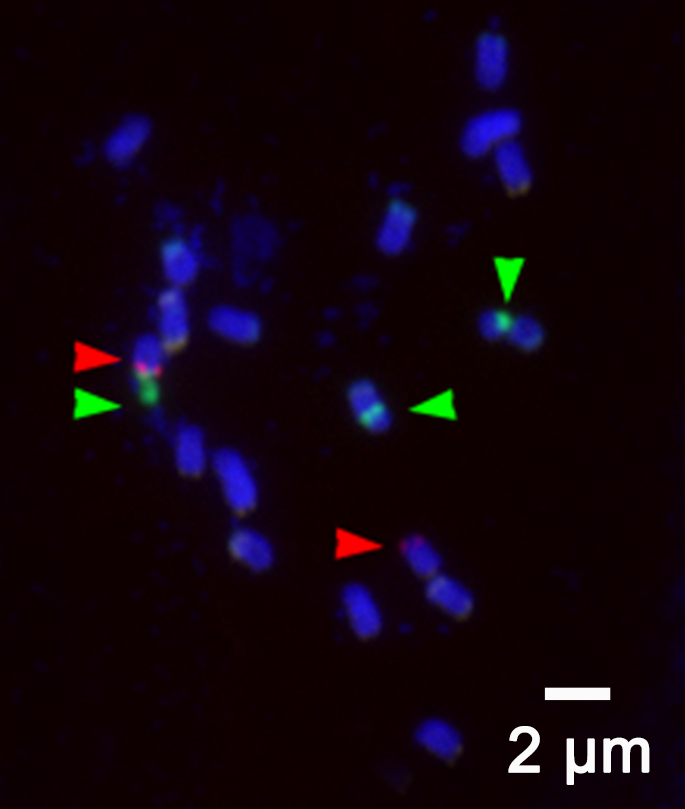

Supplement: FIGURE S2 — Distribution of 45S and 5S rDNA on the somatic metaphase chromosomes of blood orange using multicolor FISH. The green arrowheads indicate the 45S rDNA loci (green signals), and the red arrowheads indicate the 5S rDNA loci (red signals). [file Image_2.TIF]
